# Complex Pulmonary Artery Rehabilitation in Children with Alagille Syndrome: An Early Single-Center Experience of a Successful Collaborative Work

**DOI:** 10.3390/jcdd11080232

**Published:** 2024-07-25

**Authors:** Farida Karim, Gurumurthy Hiremath, Juan Carlos Samayoa, Sameh M. Said

**Affiliations:** 1 Division of Pediatric Cardiology, Masonic Children’s Hospital, University of Minnesota, Minneapolis, MN 55454, USA; karim166@umn.edu (F.K.); hiremath@umn.edu (G.H.); samay008@umn.edu (J.C.S.); 2 Division of Pediatric and Adult Congenital Cardiac Surgery, Department of Surgery, Maria Fareri Children’s Hospital, Westchester Medical Center, New York Medical College, Valhalla, NY 10595, USA; 3 Department of Cardiothoracic Surgery, Faculty of Medicine, Alexandria University, Alexandria 21544, Egypt

**Keywords:** Alagille syndrome, branch pulmonary artery stenosis, pulmonary artery branch rehabilitation, liver failure, right ventricular outflow tract obstruction, pulmonary arterial branch reconstruction

## Abstract

*Objective:* In this paper, we share our single-center experience of successful multidisciplinary management of patients with Alagille syndrome. In addition, we aim to highlight the need for an Alagille program for effectively managing these patients, in general, and particularly peripheral pulmonary artery stenosis associated with this syndrome. *Study Design:* This is a retrospective review of six children with Alagille syndrome and advanced liver involvement who underwent pulmonary artery reconstruction between 2021 and 2022. Cardiac diagnosis, co-existing liver disease burdens, management approach, and short-term outcomes were analyzed. *Results:* All the patients underwent one-stage extensive bilateral branch pulmonary rehabilitation. Concomitant procedures included repair of tetralogy of Fallot in one patient and repair of supravalvar pulmonary artery stenosis in two. One patient had balloon pulmonary branch angioplasty before surgery. In all patients, there was a decrease in right ventricular systolic pressure post-operatively. Three patients underwent liver transplantation for pre-existing liver dysfunction. At a median 3-year follow-up, all the patients were alive with their right ventricular systolic pressure less than half of their systemic systolic pressure. One patient underwent balloon angioplasty due to new and recurrent left pulmonary artery stenosis 13 months after surgery. *Conclusion:* Pulmonary arteries can be successfully rehabilitated surgically in the presence of complex branch disease. Patients with advanced liver disease can undergo successful complex pulmonary artery reconstruction, which can facilitate their future liver transplantation course. A multidisciplinary team approach is a key for successful management of Alagille patients.

## 1. Introduction

Alagille syndrome (ALGS) is a rare genetic multi-organ disease with frequent cardiovascular and hepatic system involvement. Other abnormalities include renal, ocular, neurological, facial, and/or skeletal defects. The majority of patients presented with symptoms in infancy and require a comprehensive multidisciplinary team evaluation. Due to the complex nature and interactions between the various organ systems, these patients present distinctive challenges in terms of management and timing of interventions if any are needed.

Congenital heart disease (CHD) has been reported in up to 90% of patients with ALGS [1]. The cardiovascular malformations range from peripheral pulmonary artery stenosis (PPAS) to Tetralogy of Fallot (TOF) with pulmonary atresia and major aortopulmonary collateral arteries (MAPCAs) [2]. Hepatic involvement in ALGS also has an extremely variable expression. It can range anywhere from mild cholestasis and paucity of the intrahepatic bile ducts to progressive liver failure. It is estimated that half of all patients with ALGS will eventually require liver transplantation (LTx) [3].

The subset of ALGS patients with concomitant complex CHD and liver disease is by far the most difficult to manage. One of the important considerations is the timing of cardiovascular repair in patients who develop advanced liver dysfunction with the potential need for LTx. There is often a reluctance to perform LTx in the setting of unrepaired CHDs, particularly when they involve right heart structures due to the presence of an elevated right ventricular/systemic venous pressure, which could compromise the outcomes or increase the risk of liver transplant. On the other hand, performing a complex cardiac operation on a patient with advanced liver disease significantly increases the peri- and post-operative risks and morbidity/mortality of the cardiac surgical procedure. Mainwaring et al. reported a four-fold higher relative risk of mortality compared to patients with mild or no liver dysfunction due to several reasons, such as sepsis, multisystem organ failure, massive pulmonary hemorrhage, and progression of liver disease [3].

Another challenge is the lack of consensus regarding the best management approach for PPAS. PPAS is found in less than 1% of patients with CHD [4]. Current practices vary from extensive surgical pulmonary artery (PA) reconstruction to transcatheter interventions. However, the goal of any of these interventions should be to effectively normalize right ventricular (RV) pressure and provide long-term outcomes with low morbidity, mortality, and acceptable freedom from re-intervention for these patients [5].

The purpose of this article is to highlight the need for developing ALGS programs based on a multidisciplinary group of subspecialties to address the unique needs of this challenging patient cohort. Through our multidisciplinary ALGS team, we were able to produce the following satisfactory results, which represent our early center’s multidisciplinary experience.

## 2. Materials and Methods

### 2.1. Patient and Procedure Characteristics

This is a single-center retrospective review of 6 pediatric patients (<18 years) with a confirmed diagnosis of ALGS, who underwent extensive bilateral pulmonary arterial branch rehabilitation. The patient’s baseline characteristics (Table 1), surgical procedure details, peri-operative outcomes, and follow-up were reported. We summarized variables using median and range where specified; we reported counts for categorical variables. The Institutional Review Board at the University of Minnesota approved this study (STUDY ID 00017269, approval date: 10 October 2022).

### 2.2. Multidisciplinary Team Evaluation

Given the potential for multisystem involvement in ALGS, all 6 patients underwent a thorough multidisciplinary evaluation that included expertise in genetics, gastroenterology, hepatology, pulmonology, and hematology before any interventions. In particular, the hematologic workup included detailed assessments for thrombotic and bleeding risks to plan the peri-operative management of these patients carefully.

Of note, two out of the six patients were referred to cardiology from gastroenterology for LTx clearance due to pre-existing end-stage liver disease (ESLD) and were found to have concomitant PPAS. Patients with pre-existing liver involvement were risk-stratified by the best management strategy and timing of LTx in relation to the PPAS repair. Both patients with pre-existing ESLD were deemed to be at high risk for LTx without PPAS repair due to significantly elevated RVSP secondary to the multilevel right ventricular outflow tract obstruction.

In addition, the PELD (Pediatric End-Stage Liver Disease) score within one month before the cardiac repairs was used for risk stratification of the remaining four patients. PELD determines prognoses and prioritizes recipients for LTx, for whom the higher the PELD score, the more severe the liver dysfunction [6]. Additional pre-operative discussion involved the critical care, cardiology, pediatric cardiac anesthesia, and pediatric cardiac surgical teams.

### 2.3. Technical Aspects of Branch Pulmonary Artery Surgical Rehabilitation 

**Figure 1:**
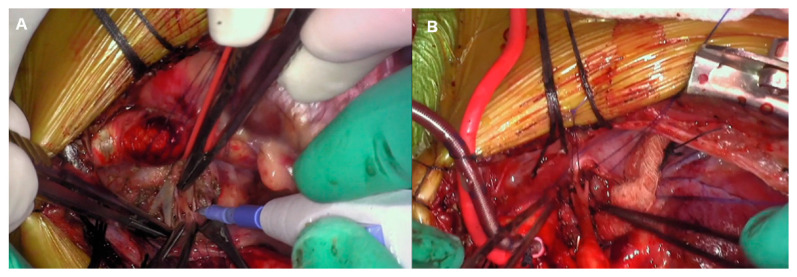
(**A**,**B**) Intra-operative photos showing (**A**) the thoroughly dissected lobar and segmental branches of the right and (**B**) left pulmonary arteries. This is an important step that has to be performed prior to heparinization and initiation of cardiopulmonary bypass to avoid the potential for pulmonary hemorrhage from the parenchymal dissection.

**Figure 2:**
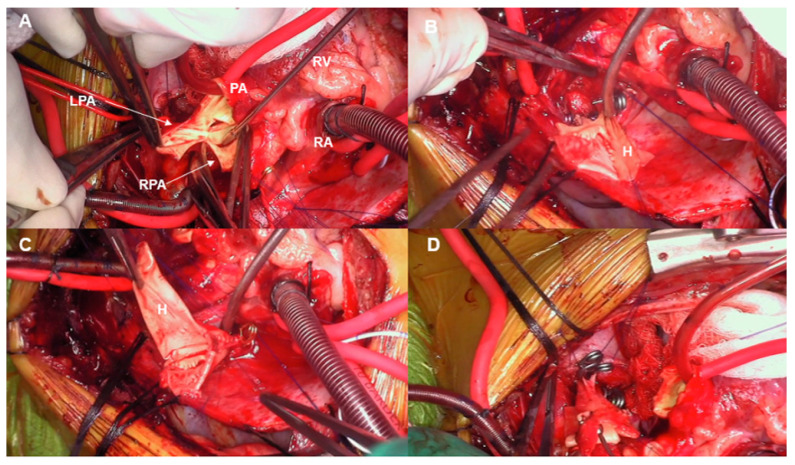
(**A**–**D**) Intra-operative photos showing (**A**) the main pulmonary artery (PA) and both branches being transected which is an important step to facilitate the dissection and reconstruction. (**B**) Decellularized pulmonary homograft (H) patch augmentation of the right pulmonary artery is being performed, and the completed reconstruction is shown in (**C**). The procedure is then repeated on the left side, as shown in (**D**). LPA: left pulmonary artery; RPA: right pulmonary artery; PA: main pulmonary artery; RV: right ventricle; RA: right atrium, H: homograft.

**Figure 3:**
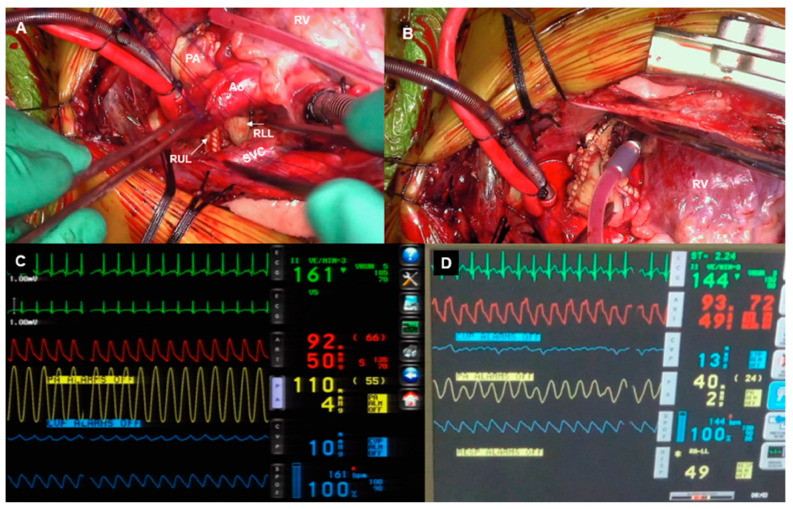
(**A**–**D**) Intra-operative photos showing the final appearance of the augmented right (**A**) and left (**B**) branch pulmonary arteries. Pressure tracings showing (**C**) pre-cardiopulmonary bypass direct measurement of the right ventricular pressure (yellow tracing) which is suprasystemic, and (**D**) immediate post-operative right ventricular pressure which is less than 50% of the systemic pressure. All measurements were through a direct right ventricular pressure line that was placed intra-operatively. Ao: ascending aorta; PA: main pulmonary artery; RV: right ventricle; SVC: superior vena cava; RUL: right upper lobar branch; RLL: right lower lobar branch.

All the patients underwent one-stage midline bilateral branch PA rehabilitation. We have described the details of our procedure previously [7]. Briefly, after median sternotomy, an extensive bilateral branch PA dissection is performed before systemic heparinization (Figure 1A,B). All areas of anatomic concerns must be identified and prepared before the initiation of a cardiopulmonary bypass (CPB). The extent of dissection depends on a thorough understanding of the pre-operative imaging studies and cardiac catheterization. CPB is then initiated with a single arterial and venous cannula, and the patients are cooled to 28 °C. The initial steps require transection of the main pulmonary artery above the sinotubular junction and separation of the main PA branches (Figure 2A). This is an important step for facilitating further dissection of the distal lobar/segmental branches. The right PA is then brought from behind the ascending aorta, either in between the ascending aorta and the superior vena cava (SVC) or lateral to the SVC, and stay sutures are placed to assist with retraction and branches’ exposure (Figure 2B). This allows further distal mobilization of the branches and allows the surgeon to reach the segmental-level branching (Figure 2C). The left branch is handled in a similar manner (Figure 2D). The preferred patch material is a decellularized pulmonary homograft patch, which is used to augment and relieve all stenotic branch segments. Once we are satisfied with the degree of augmentation of the pulmonary arteries, the right pulmonary artery is brought back behind the ascending aorta in its orthotopic position and anastomosed to the main/left PA (Figure 3A,B). If no associated procedures need to be completed, the patient is gradually rewarmed and weaned off CPB in the standard fashion. We routinely leave a right ventricular pressure line in the immediate post-bypass and post-operative period. 

In the patient who requires repair of tetralogy of Fallot, once the pulmonary arterial reconstruction is completed, the SVC and inferior vena cava are cannulated. Cardioplegic arrest is then achieved, and intracardiac repair is performed in the standard fashion. A decision is then made regarding delayed sternal closure based on the ability to achieve satisfactory hemostasis or not. The detailed surgical procedures are summarized in Table 2.

## 3. Results

The present study summarizes our surgical experience with six consecutive ALGS patients who had severe PPAS causing right ventricular hypertension with systemic/suprasystemic right ventricular systolic pressure (RVSP) and underwent complete cardiac repair (Figure 3C,D). The median age and weight at the time of surgery were 20.5 (7–18) months and 9 (4.9–10) kg, respectively. The baseline characteristics and details of cardiovascular abnormalities are summarized in Table 1 and Table 2, respectively. One patient underwent transcatheter balloon angioplasty of the left PA and multiple bilateral segmental branches before surgery but had a persistent elevation in her RVSP. 

Table 3 demonstrates the immediate hemodynamic and post-surgical outcomes. All six patients showed a drastic drop in their RVSP immediately post-operatively. The median number of patches used for the pulmonary arthroplasties was five (range 2–7). The median CPB time for the entire cohort was 459 min (range 192–409 min). 

There was no peri-operative mortality. Three patients (50%) required delayed sternal closure. No peri-operative stroke or heart block required a pacemaker. The median length of hospital stay for the entire cohort was 16 days (range 7–168 days). One patient underwent LTx during the same admission. The median duration of follow-up was 20 months (max. 26 months). 

### 3.1. Liver Disease Burden

All six patients had advanced involvement of the hepatobiliary system as evidenced by high pre-operative PELD scores, and three patients underwent LTx due to ESLD at one period during this study. 

The three patients who underwent LTx had much higher pre-operative PELD scores compared to the rest of the group. Liver transplantation occurred in one patient one month after PA reconstruction and prior to hospital discharge. Two other patients’ LTx occurred at 12 and 15 months post-PA reconstruction. 

### 3.2. Follow-Up and Late Outcomes

Table 4 describes the late hemodynamic analysis and late post-surgical outcomes. Only one patient, with tetralogy of Fallot, needed transcatheter intervention (balloon dilation of recurrent stenosis of the left PA and segmental branches of the left lower lobe) 13 months later. 

**Figure 4:**
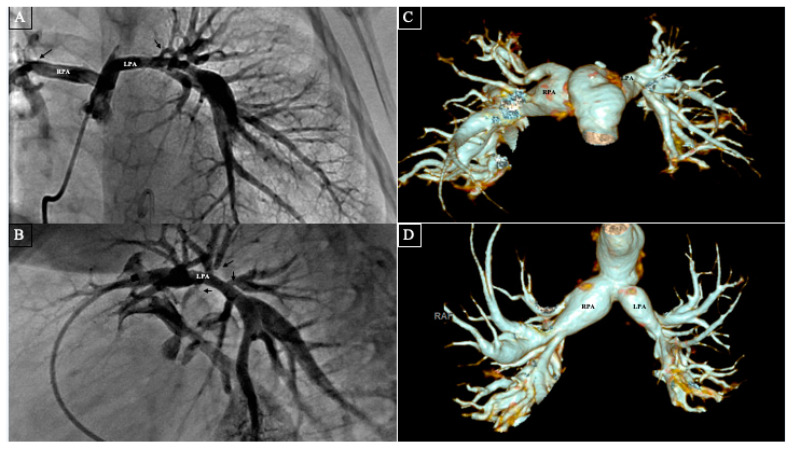
(**A**–**D**) Pre-operative pulmonary angiography: anteroposterior (**A**) and lateral (**B**) views showing severe central branch pulmonary arteries stenoses and hypoplasia. The left and right main branches measured about 3 mm and 4.5 mm, respectively which were quite small for the patient. Multiple segmental stenoses of the lobar branches can be seen as well (black arrows). Post-operative computerized tomographic 3D reconstruction images (**C**,**D**) on the same patient performed 17 months after the pulmonary arterial rehabilitation showing widely patent and much larger central branch pulmonary arteries with minimal residual segmental pulmonary artery branch stenosis. RPA: Right pulmonary artery, LPA: Left pulmonary artery.

Overall, surveillance computed tomography (CT) scans showed improved overall caliber of the branch pulmonary arteries in all the patients during their follow-up (Figure 4A–D). RVSP for all the patients remained less than 50% of the systemic arterial pressure during the follow-up period. One patient underwent kidney transplantation 17 months later due to progression to end-stage renal disease. There was no late mortality or re-operations.

## 4. Discussion

This single-center study represents an early experience with six patients who had a confirmed diagnosis of ALGS (Table 5) and underwent extensive surgical reconstruction of their PA branches. All the patients shared pre-operative systemic/suprasystemic RVSP and advanced liver disease. 

During a follow-up period of 2.2 years, all the patients underwent a satisfactory complete repair with a significant drop in their RVSP pressure, relatively balanced pulmonary blood flow, and without the need for re-intervention, apart from one patient as described previously. Outcomes were particularly favorable in patients with isolated branch pulmonary artery stenosis, given no mortality, no re-intervention, and a late follow-up pulmonary artery pressure of less than one-half systemic in most cases. These findings are consistent with the results by Leong et al. who also report low post-operative RSVP and overall favorable surgical outcomes [2].

PPAS associated with ALGS affects both the central and peripheral PA and can be extensive with varied complexity. The main physiologic consequence of PPAS is right ventricular hypertension, leading to significant hypertrophy and diastolic dysfunction. Monge et al. described the many different types of stenoses in this particular pathology, which include discrete stenoses located at the ostia or in the mid-vessel and long and tubular conformations [4]. They further reported that in their surgical experience, using homograft patch augmentation applies to all types of stenoses and effectively relieves the obstruction and effective relief of RV hypertension. Understanding these different patterns from pre-operative cardiac catheterization cross-sectional imaging is critical as it guides the surgeon in the operating room and ensures proper repair of these stenotic areas. We have used the same patch material in the current study as we believe it gives satisfactory long-term results and does not calcify or result in significant scarring in this particular location compared to other patch materials available. 

The optimal treatment strategy for PPAS associated with ALGS continues to be debated among centers advocating either surgical or percutaneous PA interventions [5]. The reason for this is the rarity of both ALGS and PPAS. The decision tree in these cases takes into consideration the available expertise as well as whether the surgical experience outweighs the transcatheter one or vice versa. The group at Stanford University is a major proponent of primary surgical repair of PPAS, whether it is associated with ALGS or not, and this is based on their extensive experience in pulmonary arterial branch reconstruction from tetralogy of Fallot with pulmonary atresia and MAPCAs. However, ALGS patients are more challenging in terms of their disease burden, higher chance of recurrence of stenosis, and the associated multisystem organ involvement, which further increases the peri-operative risks. Luong et al. reported their experience with 51 patients with ALGS who underwent PA rehabilitation [2]. They concluded most patients with ALGS and severe PA stenosis can undergo repair with low post-operative RVSP with 90% survival after a complete repair at a median follow-up of 7.9 years. This is also congruent with other studies reporting freedom from pulmonary artery catheter-based re-intervention after the first surgery for isolated branch PPAS of 95% and 80% at 12 and 36 months, respectively [8].

Several reports have also demonstrated outcomes with percutaneous techniques for the management of PPAS associated with ALGS [9,10]. Lan et al. described that extensive catheter-based interventions reduced the median systolic main PA pressure (94.0 to 49.6 mm Hg) by 47% [5]. Although significant, this was a much smaller improvement than reported in the surgical literature for patients with William syndrome (66% reduction; 80 to 27 mm Hg) and ALGS (61% reduction; 75 to 29 mm Hg). In addition, these surgical results were reported to persist at long-term follow-ups of 1.5 and 2.5 years, respectively. Additionally, Hosking et al. shared their experience with 74 patients with native or post-operative PA stenosis with 110 balloon angioplasty procedures, confirming the observations of other authors that due to the potential for a beneficial result with low complication risk, angioplasty should be offered as initial therapy [11]. However, they also could not define any predictive factors for success, and often, the clinical impact was found to be transient. Therefore, most data have uniformly advocated a multimodality or hybrid approach with a combination of catheter-based and surgical interventions on a case-by-case basis [12]. In our opinion, surgery and transcatheter approaches are complementary to each other. We prefer the surgical option to be considered first in the majority of these cases unless the risks outweigh the benefits. In addition, follow-ups should be meticulously planned for surveillance for the recurrence of stenosis which could also be addressed on time with transcatheter techniques.

All patients in the current study have varying degrees of advanced liver disease. This represents a challenge in ALGS patients but is often the first red herring that points out their associated PA disease burden, as outlined in two patients in the current study who were initially referred for LTx evaluation. The difficulty in making the decision in these cases comes from the known risks of performing major surgery on a patient with advanced liver disease and, on the other hand, the challenges of transplanting the liver in a patient with suprasystemic RVSP also leading to elevated central venous pressure. Mainwaring et al. reported that patients with moderate or severe liver dysfunction had a four-fold higher relative risk of mortality compared to patients with mild or no liver dysfunction [3]. They suggested that liver dysfunction has an important influence on outcomes in children with ALGS undergoing CHD surgery. We demonstrated with careful pre-operative planning and a multidisciplinary team approach, that in those with ALGS and advanced liver disease, pulmonary arterial reconstruction can be performed safely; although it may require significant post-operative work, it will smoothen the path for LTx if needed later.

Luong et al. [2] supported the notion that PA disease alone is not a contraindication to LTx in patients with ALGS and ESRD. Given the prolonged surgical and bypass times required for PA reconstruction surgery with expected unfavorable hepatic sequelae, all patients with ALGS and significant liver disease should undergo evaluation by a transplantation hepatologist and LTx surgeon before PA reconstruction surgery. This study also showed that patients with severe liver disease can undergo complex PA repair and that those with repaired PA anomalies can undergo successful LTx.

In light of the complex nature and interactions between the various organ systems involved, our report concludes that a multidisciplinary team approach is certainly needed for ALGS patients. Furthermore, there is a pressing need to establish Alagille multidisciplinary programs to risk-stratify these patients and determine the ideal approach to their overall management. 

## 5. Conclusions

Pulmonary arteries can be successfully rehabilitated surgically in the presence of complex branch stenosis with low morbidities or need for re-intervention. Patients with advanced liver disease can undergo successful complex pulmonary artery reconstruction, which can facilitate their future liver transplantation course. A multidisciplinary team approach is key to the successful management of Alagille patients due to its complex nature and frequent involvement in multisystem organs.

## 6. Limitations

The major limitation of this study is related to its small number; however, this is a relatively good number for the period of this study, considering the rarity of this disease and the rarity of having ALGS with advanced liver dysfunction. We also described early outcomes, but this was an important step for our program to highlight our approach and lessons learned with the goal of continuing to evolve our technique and highlight long-term data when available. 

## Figures and Tables

**Table 1:** Patients’ baseline characteristics.

Characteristics		Patients (n = 6)
Female sex		4
Median age at the time of cardiac surgery (months)		20.5 (7–18)
Median weight at the time of cardiac surgery (kg)		9 (4.9–10)
Positive for JAG 1 gene deletion		5 *
**Organ System Involvement**		
Gastro-intestinal involvement		6
	Median PELD score	25.6 (6.7–22.3)
	ESLD requiring liver transplantation ^¥^	3
Renal diagnosis	ESRD requiring renal transplantation ^≠^	3 1
Hematological diagnosis		2
	Complex coagulopathy ^€^	
Axial skeletal involvement		2
	Butterfly vertebrae	1
	Long bone fractures	1
Ophthalmologic involvement		3
	Posterior embryotoxon	2
	Retinal dystrophy	1

JAG 1, Jagged Canonical Notch Ligand 1; ESLD, end-stage liver disease; PELD, pediatric end-stage liver disease score; ESRD, end-stage renal disease. * One family opted out of genetic testing but has a phenotypic diagnosis of ALGS. ^¥^ Developed ESLD during the follow-up. ^≠^ Developed ESRD during the follow-up. ^€^ Global coagulation factor deficits, thrombocytopenia likely from hypersplenia, platelet dysfunction.

**Table 2:** Associated cardiovascular anomalies and the performed surgical procedures.

Cardiac Diagnosis	Patients (n = 6)
Branch pulmonary artery stenosis	6 *
Central pulmonary artery stenosis	2
Criss-cross branch pulmonary arteries	3
Supravalvar pulmonary artery stenosis	2
Pulmonary valve stenosis	1
TOF	1
Bicuspid pulmonary valve	1
ASD	4
PDA	4
Bicuspid aortic valve	1
Coarctation of the aorta	1
**Surgical Interventions**	
Extensive bilateral branch pulmonary patch arterioplasty	6
TOF repair	1
Open pulmonary valvotomy	1
Tricuspid valve repair	1
Repair of supravalvular PA stenosis	2
ASD closure	4
PDA ligation	4

TOF, tetralogy of Fallot; PA, pulmonary artery; ASD, atrial septal defect; PDA, patent ductus arteriosus. * Most patients had a combination of central and peripheral lobar and segmental PAS.

**Table 3:** Immediate hemodynamic and post-surgical outcomes.

Hemodynamics	Patients (n = 6)
**RVSP ^£^ prior to surgery**	
Suprasystemic	2
Systemic	3
>50% systemic	1
**RVSP ^£^ immediate post-op**	
Suprasystemic	0
Systemic	0
50–70% systemic	4
<50% systemic	2
**Surgical Details**	
Median cardiopulmonary bypass time (minutes)	459 (192–409)
Median number of patches for PA plasty	5 (2–7)
**Post-op complications**	
Pericardial effusion requiring catheter drainage	1
Tamponade requiring early re-operation **	1
Wound infection and superficial dehiscence	2
**Post-op course**	
Need for ECMO	0
Median length of hospital stay (days)	16.5(7–168 *)

RVSP, right ventricular systolic pressure; ECMO, extra-corporeal membrane oxygenation; PA; pulmonary artery. ^£^ Measured with direct right ventricular pressure line placed during surgery except for one patient with TOF. TOF by definition has systemic RVSP, and post-surgical RVSP was measured via trans-esophageal echo. * One patient underwent liver transplantation during the same admission. ** This was the patient who required early liver transplantation and had significant coagulopathy in the peri-operative period.

**Table 4:** Late follow-up hemodynamic and post-surgical outcomes.

Characteristics	Patients (n = 6)
**RVSP by echo/cardiac catheterization on the last follow-up visit** Suprasystemic/Systemic 75–95% systemic <50% systemic	0 0 6
**Need for percutaneous intervention after surgery ^€^**	1
**Liver transplantation** Median duration after PPAS repair, months	3 15 (1–18)
**Kidney transplantation** Duration after PPAS repair, months	1 17
**Follow-up status overall** **Deceased** **Alive** **Prescribed PH medication**	0 6 0

RVSP: right ventricular systolic pressure; PPAS: peripheral branch pulmonary artery stenosis; PH: pulmonary hypertension. ^€^ Thirteen months after the cardiac repair, with balloon dilation of the left pulmonary artery due to restenosis and segmental branches of the left lower lobe due to development of new stenoses.

**Table 5:** Details of the genetic mutations * in the current cohort **.

Patient 1	Heterozygous frameshift mutation in JAG1:NM_000214.2; c.1205dup (p.Gln403ThrfsX13)
Patient 2	Monoallelic mutation in JAG1 variant c3063_3034dupAC
Patient 3	Heterozygous frameshift mutation in JAG1: NM_000214.3; c.2323del p. (Glu775Argfs*45)
Patient 4	Monoallelic mutation in JAG1
Patient 5	Copy number LOSS in 20p12.2 (2.0 Mb)

* Genetic testing with either chromosomal microarray or next-generation sequencing. ** One family opted out of genetic testing.

## Data Availability

Data are contained within the article.
